# Comments to “Frailty is associated with hospital readmission in geriatric patients: a prognostic study”: Assessment of frailty in geriatric patients: let’s keep it simple

**DOI:** 10.1007/s41999-020-00360-9

**Published:** 2020-07-18

**Authors:** Raphael Romano Bruno, Bernhard Wernly, Bertrand Guidet, Hans Flaatten, Dylan W. De Lange, Christian Jung

**Affiliations:** 1 Department of Cardiology, Pulmonary Diseases and Vascular Medicine, Medical Faculty, Heinrich-Heine-University Duesseldorf, Moorenstraße 5, 40225 Duesseldorf, Germany; 2 Department of Cardiology, Paracelsus Medical University, Salzburg, Austria; 3 Assistance Publique-Hôpitaux de Paris, Hôpital Saint-Antoine, Service de Réanimation Médicale, Paris, 75012 France; 4 Department of Clinical Medicine, University of Bergen, Bergen, Norway; 5 Department of Anaesthesia and Intensive Care, Haukeland University Hospital, Bergen, Norway; 6 Department of Intensive Care Medicine, University Medical Center, University Utrecht, Utrecht, The Netherlands

Dear Editor,

We read with great interest, the article “Frailty Is Associated With Hospital Readmission in Geriatric Patients: A Prognostic Study” [1]. The authors assessed frailty using the Multidimensional Prognostic Index (MPI) in geriatric patients who were acutely admitted. The MPI bases on Comprehensive Geriatric Assessment (CGA), which integrates findings from different domains including social aspects, activities of daily living (ADL), but also nutritional status and individual morbidity. Patients who were graded being moderate or severe frail evidenced a higher likelihood of hospital readmission. The authors conclude that assessing frailty using the MPI will be of great value in clinical decision making.

The assessment of frailty is an essential cornerstone in the management of all acutely ill older patients. Frailty is a multi-dimensional clinical syndrome with highly diverse etiology. In our opinion, the MPI, which integrates information from distinct clinical, social, and functional assessments, mirrors the complexity of frailty. In that regard, the MPI might be considered superior to the Hospital Frailty Risk Score (HFRS), which was recently also proposed for the quantification of frailty. While the HFRS might be useful to assess morbidity, it falls short in evaluating the true burden of frailty, which consists of more than just the sum of comorbidities [2]. Here, the MPI transcends adding up diseases and—integrating multiple dimensions of the patient’s daily life—provides a better picture. Still, it is well known that readmission rates often increase with age. In fact, the patients in the very frail MPI-group were significantly older than the non-frail group (84.9 years versus 81.8 years).

However, the fact that MPI bases on a CGA is Janus faced. On the one hand, it likely improves the clinician’s understanding of the patient. On the other hand, it depends on a multi-dimensional assessment warranting for a multi-disciplinary approach. Although this seems reasonable having frail old patients, such a time- and resource-intensive approach might not be feasible in acutely ill patients. Here, the Clinical Frailty Scale (CFS) could be the sweet spot, satisfying both the need for a thorough multi-dimensional patient assessment as well as the need for time efficiency. The CFS was already successfully applied in large clinical trials on older patients by our group [3–5]. One of the most important findings was that assessing ADL and counting comorbidities did not improve the predictive value of the CFS [4]. In other words: increasing the time and resources for frailty does not lead to improved prognostic accuracy.

In current times where resources might be limited and time-critical decision making is needed, keep frailty-assessment not only accurate but also simple.
